# Is Total Hip Arthroplasty Feasible in Patients with Severe Preoperative Thrombocytopenia Managed by a Structured Perioperative Protocol? A Propensity Score-Matched Cohort Study

**DOI:** 10.3390/jcm15156109

**Published:** 2026-08-06

**Authors:** Jong Min Park, Jae Hak Lim, Ji Hoon Bahk, Woo Lam Jo, Saad Mohammed AlShammari, Young Wook Lim

**Affiliations:** 1 Department of Orthopedic Surgery, Gadget Hospital, Gwangju 61665, Republic of Korea; pjm8732@hanmail.net; 2 Department of Orthopedic Surgery, Seoul St. Mary’s Hospital, College of Medicine, The Catholic University of Korea, Seoul 06591, Republic of Korea; wogkr66@naver.com (J.H.L.); tonybahk@gmail.com (J.H.B.); jis25@naver.com (W.L.J.); 3 Department of Orthopaedic Surgery, King Abdulaziz Air Base Hospital, Ministry of Defence, Dhahran 34641, Saudi Arabia; s3dmohammed90@gmail.com

**Keywords:** total hip arthroplasty, thrombocytopenia, perioperative complications, platelet transfusion, perioperative management, patient blood management

## Abstract

**Background/Objectives***:* Total hip arthroplasty (THA) routinely produces perioperative blood loss of 800–1200 mL, which raises concern that patients with thrombocytopenia may be at substantially higher surgical risk. Evidence on THA safety in this population, however, remains scarce. We compared perioperative and functional outcomes of THA between patients with preoperative platelet counts below 80 × 10^9^ cells/L and propensity score-matched controls with normal counts. **Methods***:* In this retrospective matched cohort study, we reviewed 972 consecutive primary THAs. From this cohort, 30 patients with baseline platelet counts below 80 × 10^9^ cells/L were identified and matched 1:2 with 60 control patients who had normal platelet counts (≥150 × 10^9^ cells/L) using 1:2 nearest-neighbor matching. For all patients in the thrombocytopenic group, platelet counts were corrected to above 80 × 10^9^ cells/L prior to surgery; thus, the study evaluates outcomes after preoperative hematologic optimization rather than surgery performed at uncorrected thrombocytopenic levels. All patients were closely monitored for at least one week postoperatively to evaluate complications and clinical outcomes. **Results***:* Operative time (78.2 vs. 78.8 min, *p* = 0.898), estimated blood loss (492 vs. 505 mL, *p* = 0.821), hematoma rate (6.7% vs. 1.7%, *p* = 0.257), surgical site infection (6.7% vs. 1.7%, *p* = 0.257), and Harris Hip Scores (86.1 vs. 85.7, *p* = 0.615) did not differ significantly between groups, although the absolute wound-complication rates were numerically about four-fold higher in the thrombocytopenic group and the small sample limited statistical power. The study group did require more packed red blood cell transfusions (7.43 vs. 1.4 units, *p* < 0.001) and had longer hospital stays (9.8 vs. 6.0 days, *p* = 0.017). **Conclusions***:* When severe preoperative thrombocytopenia is corrected to above 80 × 10^9^ cells/L and managed with a structured perioperative protocol—meticulous intraoperative hemostasis and close postoperative surveillance for re-bleeding—THA can be performed with acceptable wound complication rates and functional outcomes and without catastrophic hemorrhage. Transfusion requirements and hospital stay remain higher, and the small sample size precludes concluding that the risk of infrequent complications is equivalent to that of patients with normal platelet counts. THA is therefore feasible, rather than unequivocally safe, under careful hematologic optimization.

## 1. Introduction

Total hip arthroplasty (THA) has been called the operation of the century [1], and its demand shows no sign of slowing. Recent projections estimate a 176% increase in primary THA among Medicare patients by 2040 [2]. One factor that makes THA unique among elective procedures is the magnitude of blood loss involved. Perioperative blood loss during THA commonly reaches 800–1200 mL [3], placing substantial demands on the hemostatic system. When hemostasis fails, the consequences can be severe—prolonged hospitalization, unplanned re-intervention, and even life-threatening hemorrhage [4]. For this reason, the hemostatic capacity of a patient should be regarded as a key determinant of surgical risk.

Thrombocytopenia—broadly defined as a platelet count below 150 × 10^9^ cells/L—can result from a range of conditions: immune thrombocytopenia (ITP), aplastic anemia, leukemia, and chronic liver disease including cirrhosis [5,6,7]. Many of these patients eventually develop osteonecrosis of the femoral head, either from the disease itself or from prolonged corticosteroid and immunosuppressant use, and they ultimately require THA [8]. Herein lies the clinical dilemma: THA already entails considerable blood loss, and operating on patients whose hemostatic reserve is compromised would seem to invite a much higher rate of perioperative bleeding complications [3,9,10].

The 2025 AABB and ICTMG international clinical practice guidelines recommend prophylactic platelet transfusion when the platelet count falls below 50 × 10^9^ cells/L before major non-neuraxial surgery, but this remains a conditional recommendation based on low- to very low-certainty evidence [11]. In fact, the foundational data behind this threshold come from a single retrospective series of 167 operations in acute leukemia patients—mostly general surgical cases—published nearly four decades ago [12]. Whether the same cutoff applies to orthopedic surgery, where extensive bone cutting and soft-tissue dissection impose a very different hemostatic burden, is far from settled. There is growing evidence that higher thresholds may be needed: Varady et al. found that a preoperative platelet count below 100 × 10^9^ cells/L was an independent risk factor for complications after hip fracture surgery [13], and Malpani et al. showed that both abnormally low and abnormally high platelet counts predicted adverse outcomes after elective THA [10]. Given the critical need to prevent epidural hematomas during neuraxial anesthesia, a technique commonly employed in THA, anesthesiology guidelines often recommend a more conservative lower limit of 70–80 × 10^9^/L [14].

Despite its clinical relevance, the question of what constitutes a safe platelet count for THA has received little attention. A handful of reports have looked at THA outcomes in isolated populations with ITP [9] or aplastic anemia [15], but to our knowledge no study has compared a broader group of thrombocytopenic patients—with platelet counts below 80 × 10^9^ cells/L—against a propensity score-matched cohort with normal counts. We therefore set out to compare perioperative and functional outcomes of THA between these two groups, with particular attention to our institutional protocol of comprehensive perioperative hematologic management. Because every thrombocytopenic patient in this cohort had the platelet count corrected to above 80 × 10^9^ cells/L before surgery, the present study should be understood as an evaluation of the safety and effectiveness of this perioperative management protocol, rather than of THA performed at uncorrected, severely thrombocytopenic baseline counts.

## 2. Materials and Methods

### 2.1. Study Design and Patient Selection

We performed a single-center retrospective cohort study at our institute. The study population comprised all consecutive patients who underwent primary THA between January 2012 and December 2024. Demographics, preoperative laboratory values, operative details, and postoperative outcomes were retrieved from our institutional database.

To be included in the study group, a patient had to meet two criteria: (1) a baseline platelet count below 80 × 10^9^ cells/L caused by a chronic condition—liver cirrhosis, ITP, aplastic anemia, leukemia, lymphoma—and (2) a platelet count that remained below 80 × 10^9^ cells/L on the day before surgery prior to correction. We excluded patients whose thrombocytopenia was transient and had resolved preoperatively, those who underwent bilateral or staged THAs during the same admission, and those with insufficient data for propensity score calculation. Thirty patients (30 THAs) ultimately met these criteria.

### 2.2. Perioperative Hematologic Management Protocol

All thrombocytopenic patients were managed according to a structured perioperative protocol developed at our institution. Before surgery, each patient received prophylactic platelet concentrate (PC) transfusions until the platelet count exceeded 80 × 10^9^ cells/L. During surgery, we paid meticulous attention to hemostasis, particularly at bleeding bone surfaces and in the soft-tissue planes. After surgery, hematologic parameters were checked immediately, then daily for two days, then twice weekly for at least one week; monitoring frequency was increased whenever a transfusion was given. Platelet concentrates were transfused if the count fell back below 80 × 10^9^ cells/L, and packed red blood cells (PRC) were given when hemoglobin dropped below 7 g/dL. The rationale for this intensive first-week surveillance was straightforward: to catch any re-bleeding episode early, before it could progress.

### 2.3. Propensity Score Matching

Because the study group differed from the general THA population in several baseline characteristics, we used propensity score matching at a 1:2 ratio to assemble a comparable control cohort. Propensity scores were estimated using a multivariable logistic regression model with low platelet status as the dependent variable and age, sex, BMI, height, and weight as covariates. One-to-two nearest-neighbor matching was performed without replacement using a caliper width of 0.2 of the standard deviation of the logit of the propensity score. The adequacy of matching was assessed using standardized mean differences (SMDs), with an absolute SMD below 0.1 considered indicative of adequate balance. From the remaining 942 patients with normal platelet counts (≥150 × 10^9^ cells/L), 60 matched controls were selected. The matching model included only demographic and anthropometric variables; comorbidity burden, baseline hematologic indices, and hepatic function were not incorporated, and residual confounding from these factors cannot be excluded.

### 2.4. Surgical Technique

Three fellowship-trained hip surgeons (with 20, 10, and 5 years of experience, respectively) performed all procedures through a modified posterolateral approach. Notably, tranexamic acid and other hemostatic agents were not used in any case; this was a deliberate decision to keep the two groups comparable. A closed suction drain was placed at the end of every procedure and removed on postoperative day 1. Weight bearing was restricted to partial, using crutches or a walker, starting on the second postoperative day and continuing for six weeks.

### 2.5. Outcome Measures

We assessed two categories of outcomes. Early perioperative variables included operative time, estimated blood loss (EBL), number of packed red blood cell units transfused, length of hospital stay, and wound complications (hematoma, surgical site infection). EBL was estimated by the attending anesthesiologist based on suction canister volume and gauze weight. For longer-term assessment, we used the Harris Hip Score (HHS) recorded at the most recent follow-up visit.

### 2.6. Statistical Analysis

All analyses were carried out using IBM SPSS Statistics version 24 (IBM Corp., Armonk, NY, USA), with propensity score matching and balance diagnostics performed in R. Continuous variables were compared using the independent t-test, and categorical variables using the Fisher exact test. Standardized mean differences were calculated to confirm covariate balance after matching. A *p*-value below 0.05 was considered statistically significant.

### 2.7. Ethical Considerations

The study protocol was approved by the Institutional Review Board of our institute (IRB approval number: KC26RISI0281) and was conducted in accordance with the Declaration of Helsinki. Because of the retrospective design, the IRB waived the requirement for individual informed consent.

## 3. Results

### 3.1. Patient Demographics

**Figure 1:**
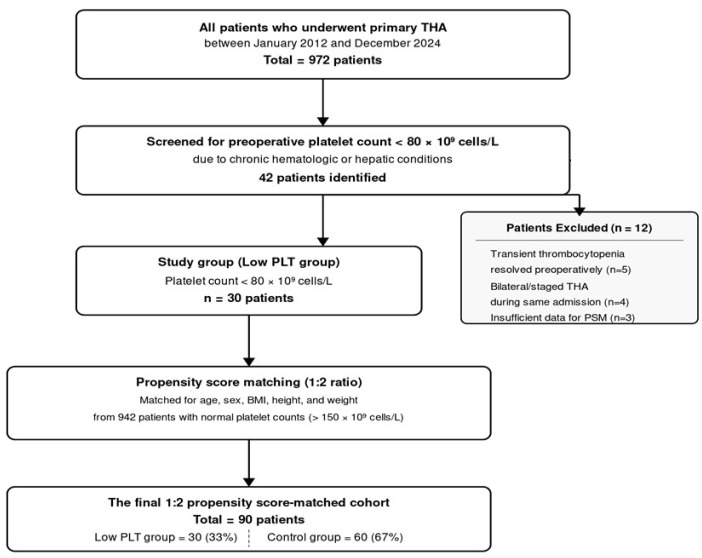
A flowchart illustrating the patient selection criteria used in this study. THA, total hip arthroplasty; PLT, platelet; PSM, propensity score matching; BMI, body mass index.

Of 972 primary THAs performed during the study period, thirty patients met the inclusion criteria (platelet count < 80 × 10^9^ cells/L). The underlying diagnoses were hypoplastic anemia (12 patients), liver cirrhosis (6 patients), diffuse large B-cell lymphoma (4 patients), acute myeloblastic leukemia (3 patients), acute lymphoblastic leukemia (2 patients), myelodysplastic syndrome (2 patients), and ITP (1 patient). Sixty patients with normal platelet counts (≥150 × 10^9^ cells/L) were matched as controls. Median follow-up was 47.5 months (range, 24–123) in the low PLT group and 64.5 months in the control group. The patient selection process is summarized in Figure 1.

Propensity score matching produced two well-balanced groups. There were no significant differences in age (47.23 ± 13.77 vs. 46.67 ± 13.13 years, *p* = 0.43), sex (male/female: 13/17 vs. 31/29, *p* = 0.485), height (161.97 ± 7.94 vs. 163.77 ± 9.15 cm, *p* = 0.264), weight (61.93 ± 11.36 vs. 63.18 ± 13.04 kg, *p* = 0.560), or BMI (23.47 ± 3.04 vs. 23.41 ± 3.70 kg/m^2^, *p* = 0.917). All standardized mean differences for the matched covariates were below 0.1, confirming adequate balance between the groups (Table 1).

### 3.2. Perioperative Outcomes

Operative time was virtually identical between the two groups (78.2 ± 12.9 vs. 78.8 ± 14.1 min, *p* = 0.898), as was estimated blood loss (492 ± 218 vs. 505 ± 195 mL, *p* = 0.821). The low PLT group did, however, require considerably more packed red blood cell transfusions (7.43 ± 5.2 vs. 1.4 ± 1.8 units, *p* < 0.001) and had a longer hospital stay (9.8 ± 6.1 vs. 6.0 ± 2.7 days, *p* = 0.017).

### 3.3. Postoperative Complications

Wound complication rates were not significantly different between groups. Wound hematoma developed in two low PLT patients (6.7%) versus one control (1.7%; *p* = 0.257). Microbiologically confirmed surgical site infection occurred in two patients (6.7%) in the study group and one (1.7%) in the control group (*p* = 0.257). Although these differences did not reach statistical significance, the absolute rates of both hematoma and SSI were approximately four-fold higher in the low PLT group; given the small sample size, the study was underpowered to detect true differences in such infrequent events, and these non-significant results should not be interpreted as evidence of equivalent risk (see Section 4, Limitations). Among the thrombocytopenic patients, three needed additional management for pancytopenia with febrile episodes, and one underwent revision surgery for recurrent hip dislocation. Of note, no catastrophic hemorrhagic event occurred in either group—an observation we attribute to the structured perioperative management protocol.

### 3.4. Functional Outcomes

Harris Hip Scores at the latest follow-up were comparable between groups (86.1 ± 8.4 vs. 85.7 ± 7.9, *p* = 0.615), indicating that the thrombocytopenic patients achieved a similar degree of functional recovery despite a more complex perioperative course. All perioperative outcomes are summarized in Table 2.

## 4. Discussion

This study demonstrates that THA can be performed with acceptable perioperative and functional outcomes in patients whose baseline platelet counts fall below 80 × 10^9^ cells/L, provided that the platelet count is corrected preoperatively and a structured perioperative hematologic management strategy is in place. It is important to emphasize that our protocol corrected all thrombocytopenic patients to above 80 × 10^9^ cells/L before surgery; accordingly, our findings reflect the outcomes of THA performed after hematologic optimization, rather than THA performed at uncorrected, severely thrombocytopenic baseline counts. Given that THA routinely produces substantial blood loss, one would reasonably expect that patients with compromised hemostatic capacity would fare worse. When platelets are corrected preoperatively, hemostasis is pursued meticulously during surgery, and re-bleeding is watched for closely during the first postoperative week, wound complication rates and functional outcomes were not significantly different from those of matched controls, although, as discussed below, the small sample size limits the strength of this comparison.

Where, then, should the platelet threshold be set? The 2025 AABB and ICTMG guidelines still recommend transfusion when the count is below 50 × 10^9^ cells/L for major non-neuraxial surgery, but they classify this as a conditional recommendation with low- to very low-certainty evidence [11]. The underlying data—a single series of 167 operations in leukemia patients, published almost 40 years ago—were derived almost entirely from general surgical cases [12]. Orthopedic surgery, with its extensive bony bleeding and wide soft-tissue exposure, poses a qualitatively different hemostatic challenge, and there is reason to think that the 50 × 10^9^ cells/L threshold may be inadequate in this setting.

At our institution, we chose to correct thrombocytopenia to above 80 × 10^9^ cells/L—deliberately higher than the AABB recommendation—based on the clinical judgment that orthopedic procedures demand more hemostatic reserve. Our outcomes appear to support that decision. We are not the only group to advocate higher thresholds: Varady et al. reported that a platelet count below 100 × 10^9^ cells/L independently predicted 30-day morbidity and mortality after hip fracture surgery [13], while Malpani et al. found that both low and high platelet counts were risk factors for adverse outcomes in elective THA [10]. In a large series of 5617 arthroplasties, Bujnowski et al. showed that patients with counts under 100 × 10^9^ cells/L needed significantly more red blood cell transfusions [16], and Wang et al. linked chronic thrombocytopenia to increased perioperative complications after primary THA and TKA [17].

That our thrombocytopenic patients required more than five times as many packed red blood cell units as controls (7.43 vs. 1.4, *p* < 0.001) was expected and is in line with prior reports. Lim et al. found a similar pattern in ITP patients undergoing THA, even though functional results were comparable [9]. We believe this heavier transfusion burden reflects several overlapping factors: the baseline coagulopathy, the additional platelet concentrates given perioperatively, and a generally lower physiologic reserve in patients with systemic hematologic disease. What matters, however, is that this extra transfusion support was associated with wound complication rates—hematoma and infection—that were not significantly different from those of controls, even though, as detailed below, the small cohort limits the power of this comparison.

The longer hospital stay in the study group (9.8 vs. 6.0 days, *p* = 0.017) deserves some explanation. It was driven not by surgical failures but by the need for extended hematologic surveillance. Three patients developed pancytopenia with febrile episodes requiring additional workup, two had wound infections treated with intravenous antibiotics, two had small hematomas observed conservatively, and one underwent cup revision for recurrent dislocation. The point is that our protocol of close monitoring for a full week after surgery allowed us to detect and address these problems before they escalated. No patient in either group suffered a catastrophic hemorrhage.

Perhaps the most reassuring finding was that intraoperative blood loss was virtually identical between groups (492 vs. 505 mL, *p* = 0.821). This suggests that once the platelet count has been corrected preoperatively, the surgeon can achieve hemostasis as effectively as in any other patient. Kim et al. and Lim et al. arrived at the same conclusion in their series of aplastic anemia patients undergoing hip arthroplasty [15,18]. The Lim et al. series is particularly relevant because it was carried out at the same institution: they performed THA in 29 patients (45 hips) with aplastic anemia using a similar transfusion protocol, and reported no excessive bleeding [18].

A further consideration concerns our decision to withhold tranexamic acid (TXA) from all patients in both groups. Our original rationale was to preserve comparability between the groups; however, we acknowledge that, because both groups followed an identical protocol, comparability would likely have been maintained even if TXA had been administered to both. TXA is now firmly established as a standard component of patient blood management in THA, reducing perioperative blood loss and transfusion requirements without increasing thromboembolic risk [3,19,20,21]. In a randomized non-inferiority trial of primary THA and TKA, both oral and intravenous TXA effectively limited calculated blood loss and minimized transfusion needs, with comparable complication rates [21]. The omission of TXA therefore represents a deviation from contemporary practice and likely contributed to the high transfusion burden, the relatively large estimated blood loss, and the prolonged hospital stay observed in both groups, and particularly in the thrombocytopenic cohort. This limits the external validity of our findings: with routine TXA use, transfusion requirements and length of stay would probably have been lower in both groups, and the absolute between-group difference might also have narrowed.

Kim et al. have recently stressed the importance of disease-specific perioperative protocols for THA patients with systemic illnesses, emphasizing medication adjustment, surgical risk stratification, and multidisciplinary teamwork [22]. We strongly agree: in thrombocytopenic patients, the orthopedic surgeon, hematologist, and anesthesiologist must work as a coordinated team. The principle that certain patient populations carry an inherently higher perioperative bleeding risk is well established. Bahk et al. showed that THA for rapidly destructive coxarthrosis from femoral head osteonecrosis resulted in greater blood loss and more transfusions than standard primary THA [23], and a recent meta-analysis by Maroun et al. confirmed an elevated complication risk when THA is performed for femoral neck fracture rather than osteoarthritis [24]. Our thrombocytopenic cohort represents yet another such high-risk group, and the results reinforce the value of prospective perioperative planning.

Functionally, the two groups were indistinguishable at last follow-up: Harris Hip Scores averaged 86.1 in the study group and 85.7 in the controls (*p* = 0.615)—both falling in the “good” category. No patient in the study group required revision for aseptic loosening or implant failure. These findings are consistent with a recent Korean multicenter study that confirmed acceptable clinical outcomes across a broad range of preoperative platelet levels [25].

This study has several limitations. First, because the thrombocytopenic cohort was small (*n* = 30), the study was underpowered to detect differences in infrequent but clinically important complications. The absolute rates of wound hematoma and SSI were approximately four-fold higher in the low PLT group (6.7% vs. 1.7% for each), and these non-significant *p*-values should not be interpreted as evidence of equivalence; rather, they likely reflect a substantial risk of a false-negative (Type II) error. Our findings should therefore be read as indicating that acceptable outcomes were achieved and that no catastrophic hemorrhage occurred, not that the complication risk is identical to that of patients with normal platelet counts. Second, propensity score matching was based only on demographic and anthropometric variables; comorbidity burden, baseline hematologic indices, and hepatic dysfunction were not included in the model, and the thrombocytopenic cohort was biologically heterogeneous (hypoplastic/aplastic anemia, liver cirrhosis, leukemia, lymphoma, MDS, and ITP). Residual confounding from these unmeasured factors and from disease heterogeneity cannot be excluded and limits disease-specific inference. Third, the deliberate omission of tranexamic acid, while intended to preserve between-group comparability, means our results may not generalize to current TXA-based practice and may overstate the transfusion burden and length of stay attributable to thrombocytopenia itself. Finally, this was a retrospective single-center study, and we did not use viscoelastic testing (TEG/ROTEM) to guide transfusion decisions—a tool that is becoming increasingly routine. As the literature on THA in patients with specific systemic conditions continues to grow—whether rheumatoid arthritis [22] or hematologic disorders [18]—the need for condition-specific perioperative guidelines becomes more apparent. Larger, prospective, multicenter studies with standardized blood management protocols, including routine TXA, will be needed to refine platelet threshold recommendations for THA.

## 5. Conclusions

Although THA entails substantial blood loss and patients with low platelet counts might be expected to experience more complications, our findings suggest that THA is feasible in this population when severe thrombocytopenia is corrected preoperatively to above 80 × 10^9^ cells/L and managed with a structured perioperative protocol of meticulous intraoperative hemostasis and close postoperative surveillance for re-bleeding. Under these conditions, acceptable wound complication rates and functional outcomes were achieved and no catastrophic hemorrhage occurred. These patients did, however, require substantially more transfusions and longer hospital stays. Because the sample size was small, the absence of statistically significant differences in infrequent complications should not be interpreted as proof of equivalent risk. Our results support the feasibility—rather than the unqualified safety—of THA in well-selected thrombocytopenic patients whose platelet counts are corrected preoperatively, and underscore the importance of a comprehensive, team-based perioperative hematologic management protocol. Larger prospective multicenter studies incorporating contemporary blood management, including routine tranexamic acid, are needed to refine platelet thresholds for THA.

## Figures and Tables

**Table 1:** Patient demographics after propensity score matching.

Variable	Low PLT Group (*n* = 30)	Control Group (*n* = 60)	*p*
Age (years)	47.23 ± 13.77	46.67 ± 13.13	0.430
Sex (M/F)	13/17	31/29	0.485
Height (cm)	161.97 ± 7.94	163.77 ± 9.15	0.264
Weight (kg)	61.93 ± 11.36	63.18 ± 13.04	0.560
BMI (kg/m^2^)	23.47 ± 3.04	23.41 ± 3.70	0.917
Underlying disease	Hypoplastic anemia 12, LC 6, DLBL 4, AML 3, ALL 2, MDS 2, ITP 1	N/A (normal platelet counts)	
Indication for surgery	ONFH 30	ONFH 60	1.0

BMI, body mass index; LC, liver cirrhosis; DLBL, diffuse large B-cell lymphoma; AML, acute myeloblastic leukemia; ALL, acute lymphoblastic leukemia; MDS, myelodysplastic syndrome; ITP, immune thrombocytopenia; ONFH, osteonecrosis of the femoral head. Data presented as mean ± standard deviation or number.

**Table 2:** Perioperative outcomes after propensity score matching.

Variable	Low PLT Group (*n* = 30)	Control Group (*n* = 60)	*p*-Value
Initial platelet count (×10^9^ cells/L)	50.3 ± 19.7 (15–78)	246.4 ± 65.6 (159–449)	<0.001
Pre-op. post-transfusion platelet count (×10^9^ cells/L)	106.3 ± 23.8 (80–160)	—	—
Operative time (min)	78.2 ± 12.9	78.8 ± 14.1	0.898
EBL (mL)	492 ± 218	505 ± 195	0.821
PRC transfusion (units)	7.43 ± 5.2	1.4 ± 1.8	<0.001
LOS (days)	9.8 ± 6.1	6.0 ± 2.7	0.017
Hematoma, n (%)	2 (6.7)	1 (1.7)	0.257
SSI, n (%)	2 (6.7)	1 (1.7)	0.257
HHS at last follow-up	86.1 ± 8.4	85.7 ± 7.9	0.615

EBL, estimated blood loss; PRC, packed red blood cell; LOS, length of hospital stay; SSI, surgical site infection; HHS, Harris Hip Score. Data presented as mean ± standard deviation or number (%). Categorical variables were compared using the Fisher exact test.

## Data Availability

The data presented in this study are available on request from the corresponding author. The data are not publicly available due to institutional privacy regulations.
